# Supplementary figures and images for: Interactomic affinity profiling by holdup assay: Acetylation and distal residues impact the PDZome-binding specificity of PTEN phosphatase
Source: PLoS One. 2020 Dec 31;15(12):e0244613. doi: 10.1371/journal.pone.0244613 (PMC7774954; doi:10.1371/journal.pone.0244613)

Supp. Fig. S1

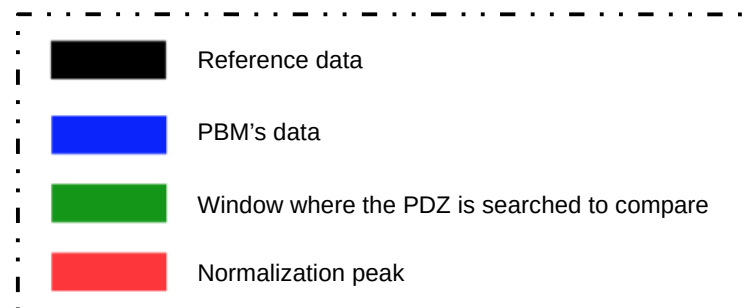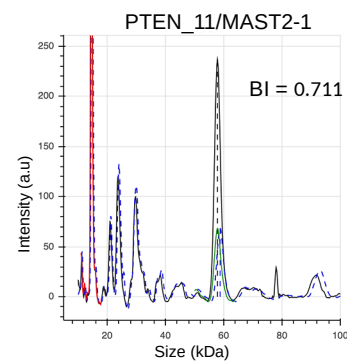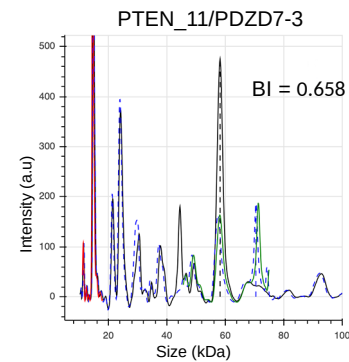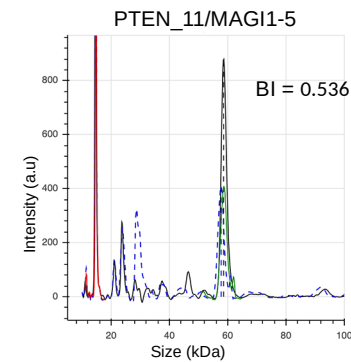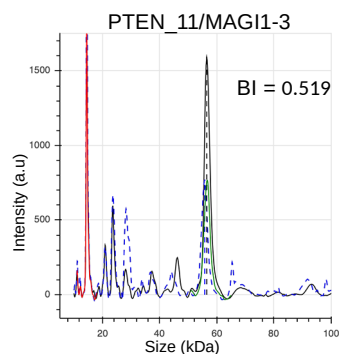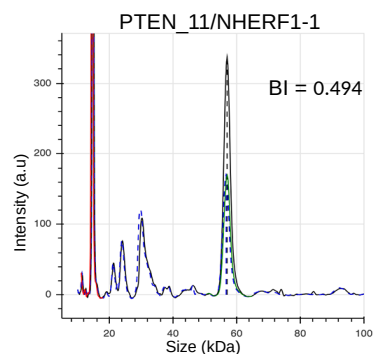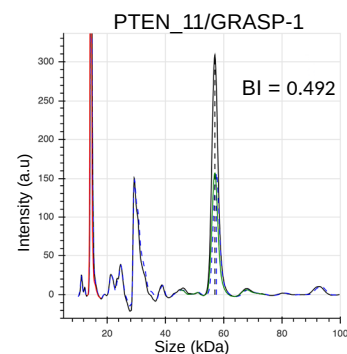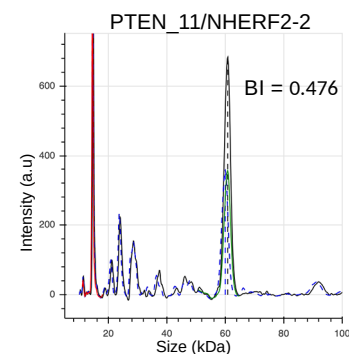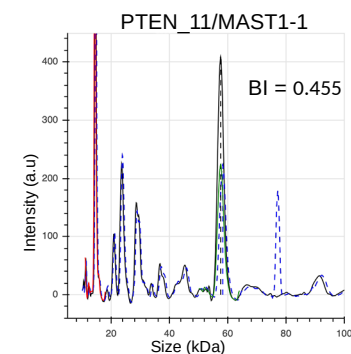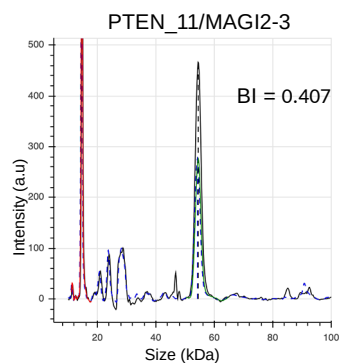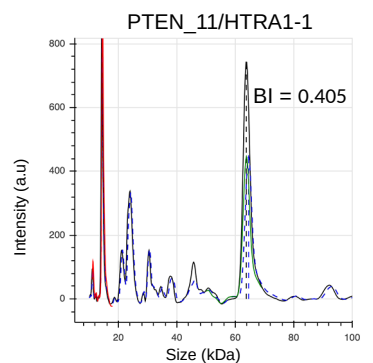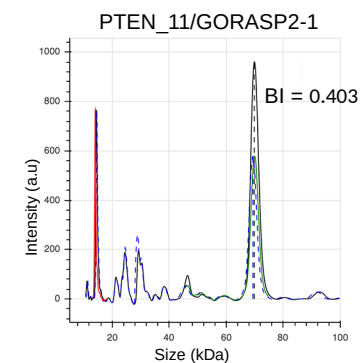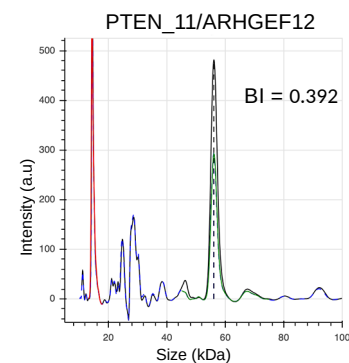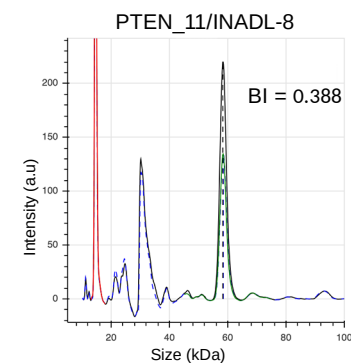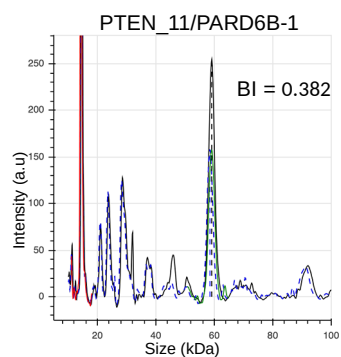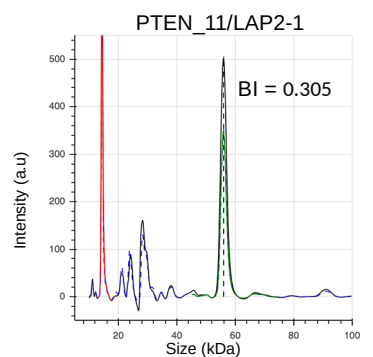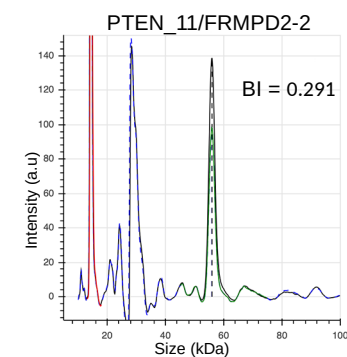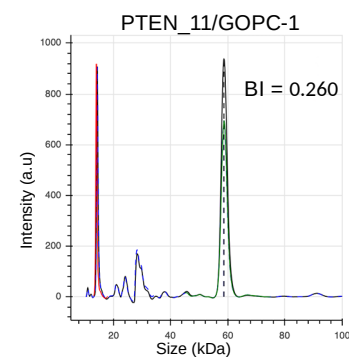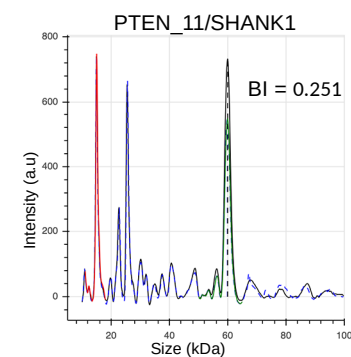

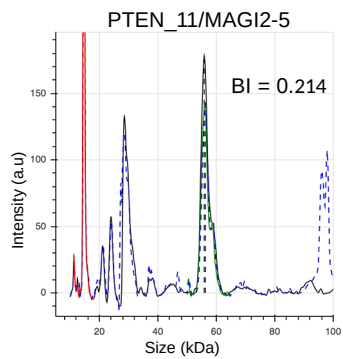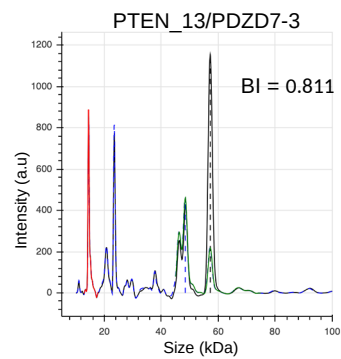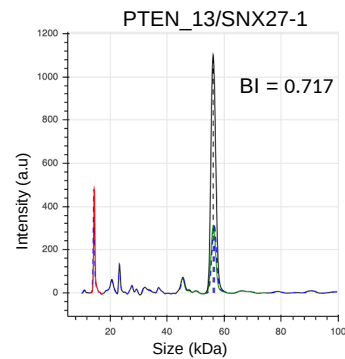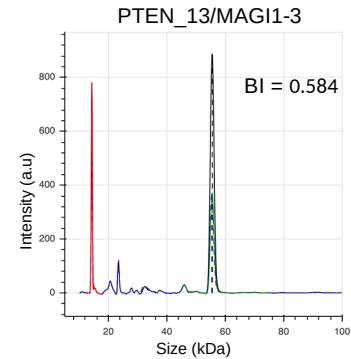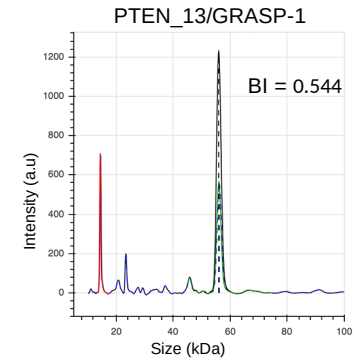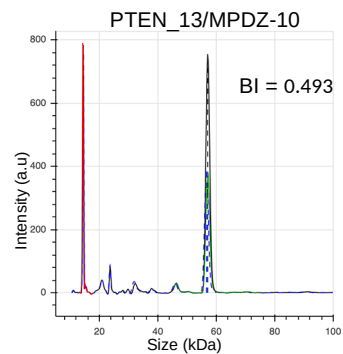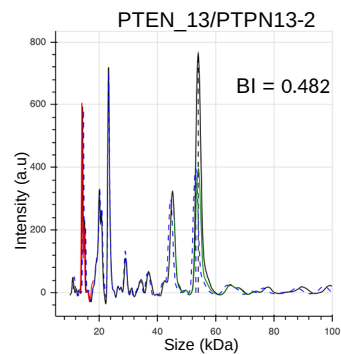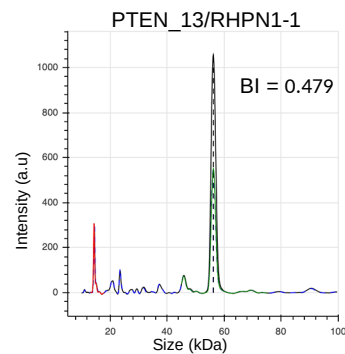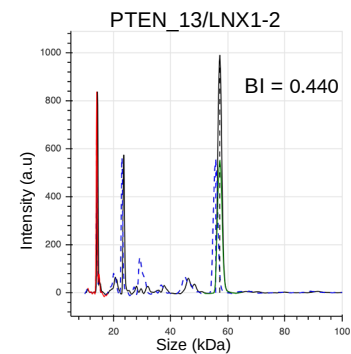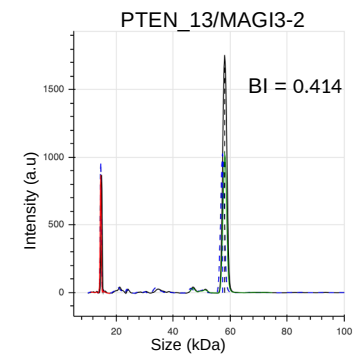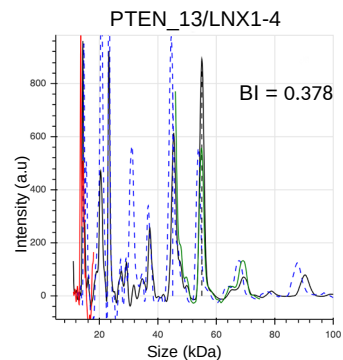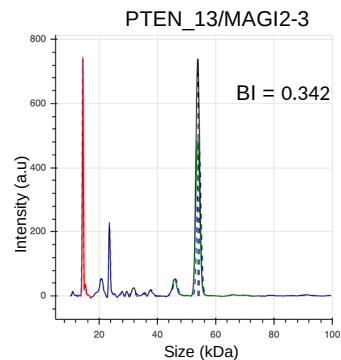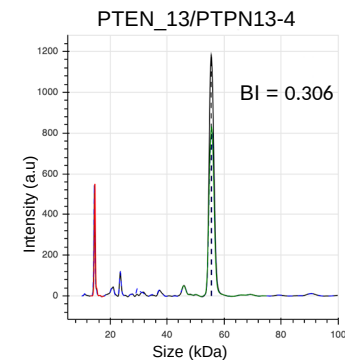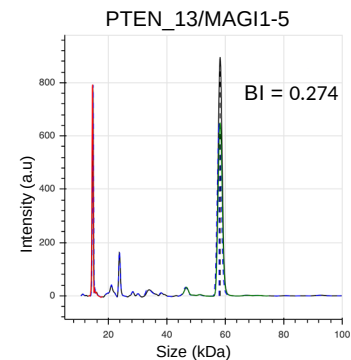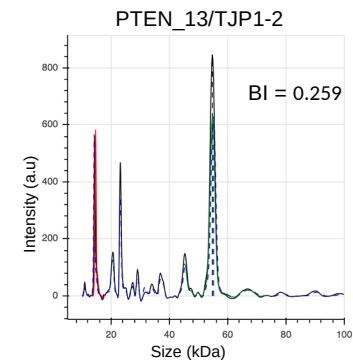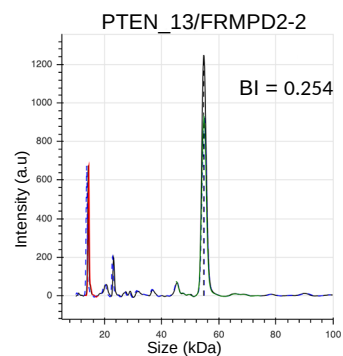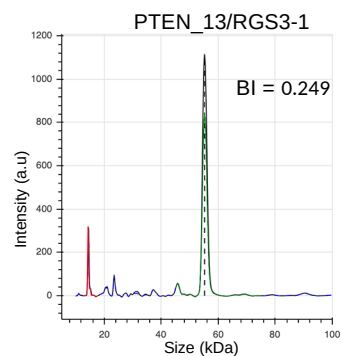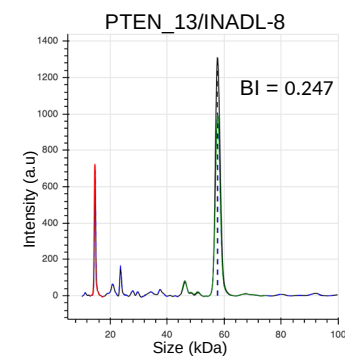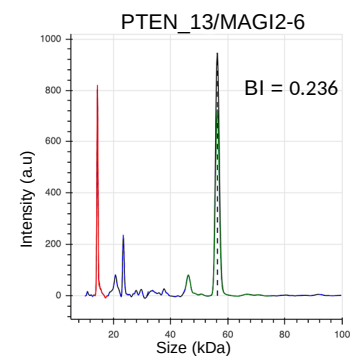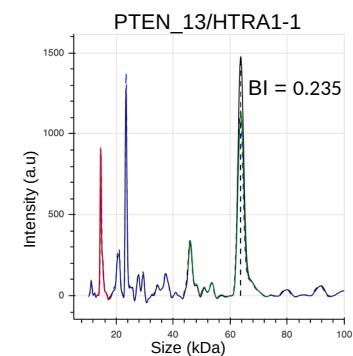

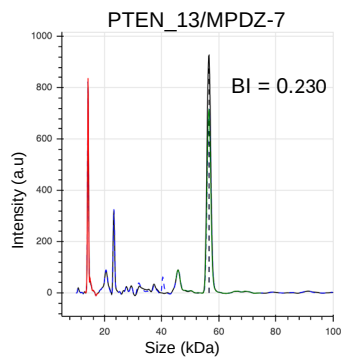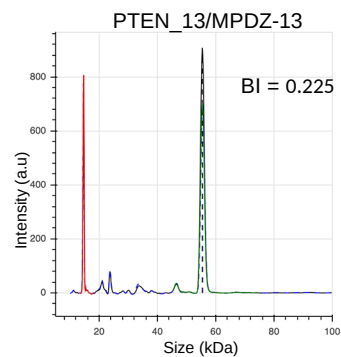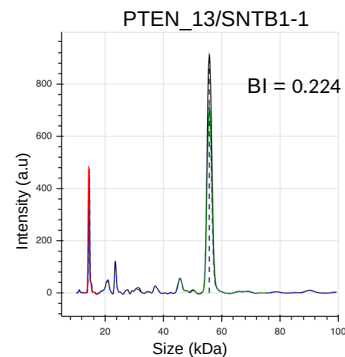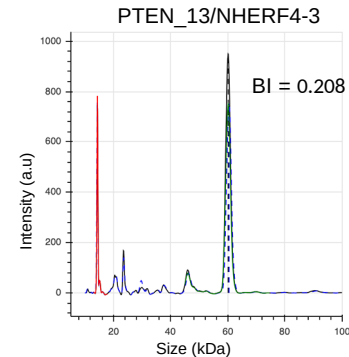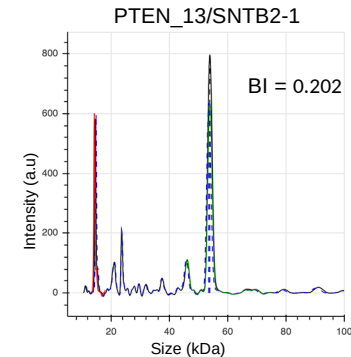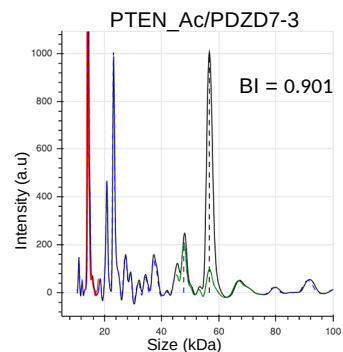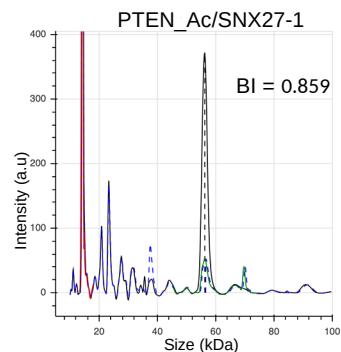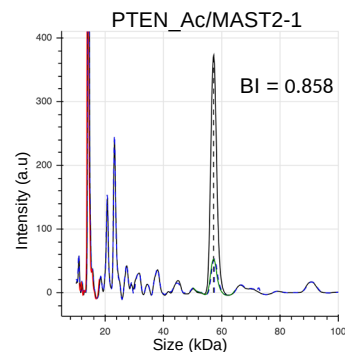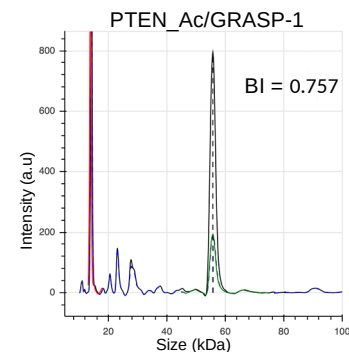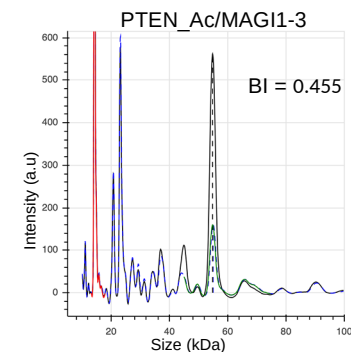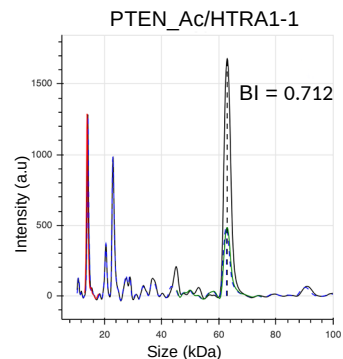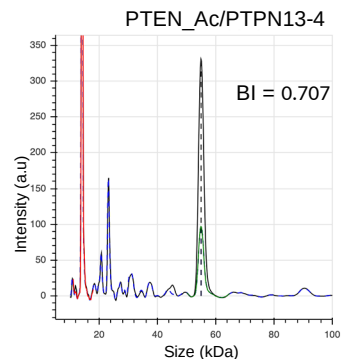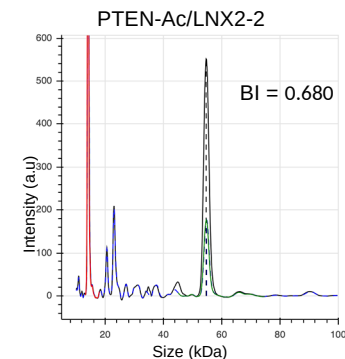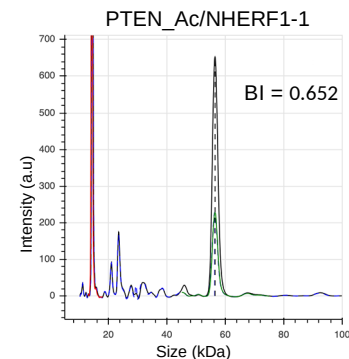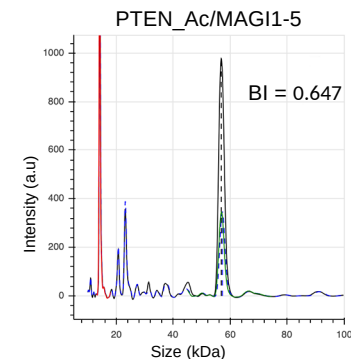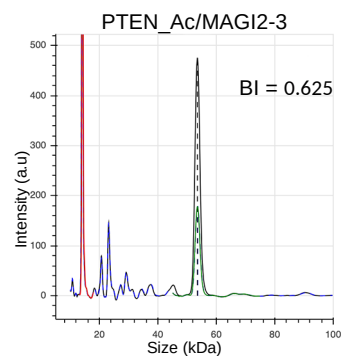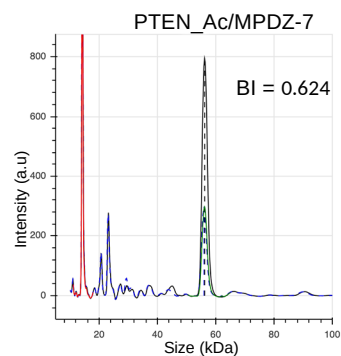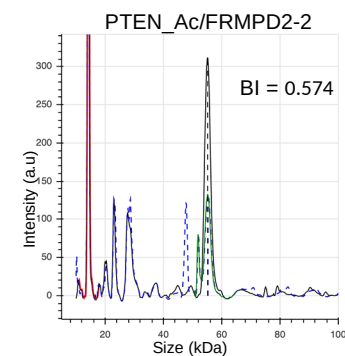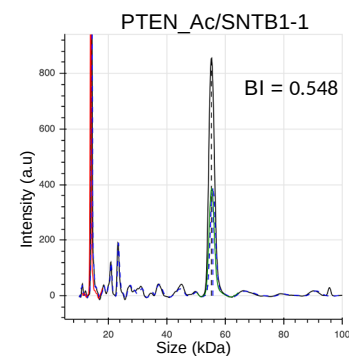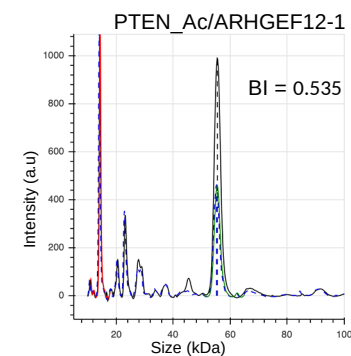

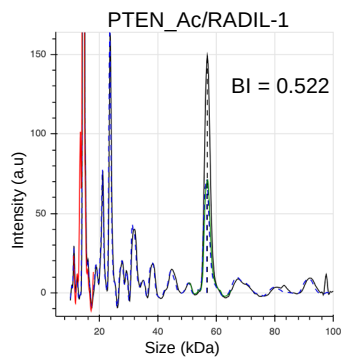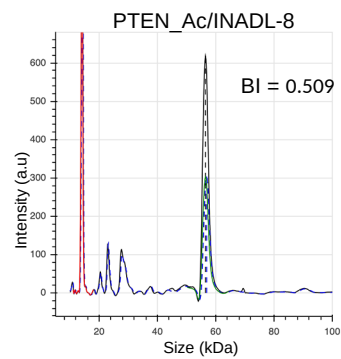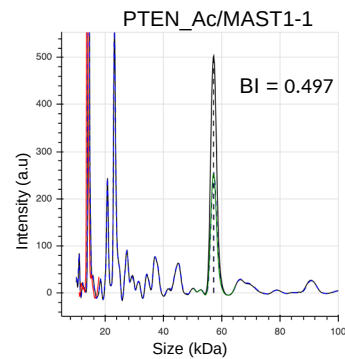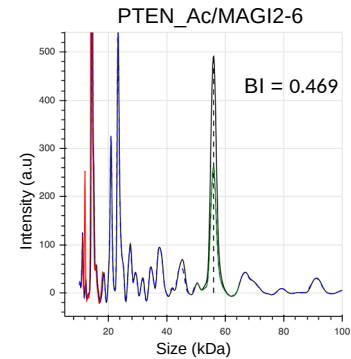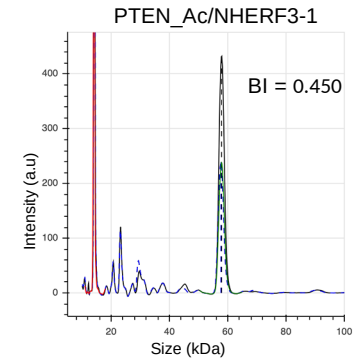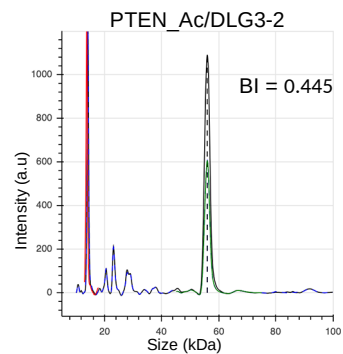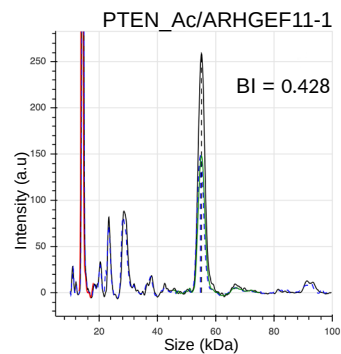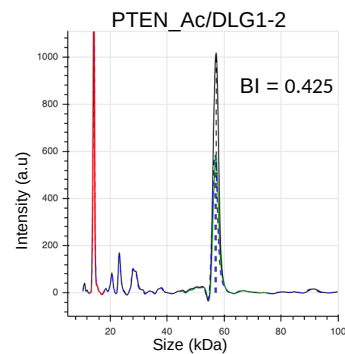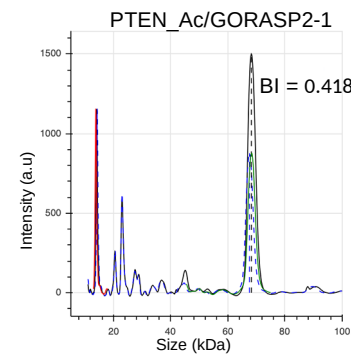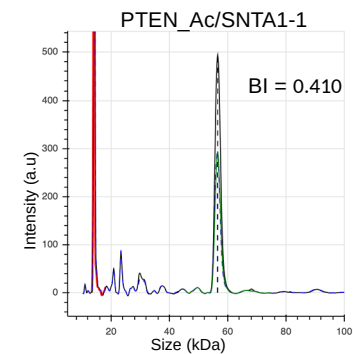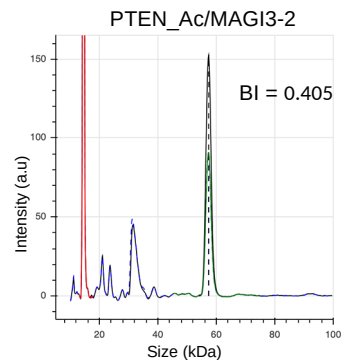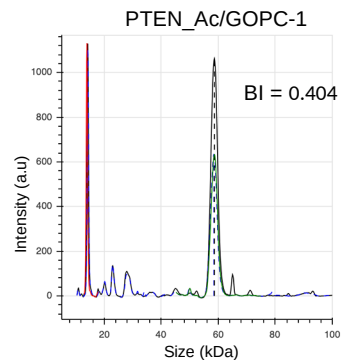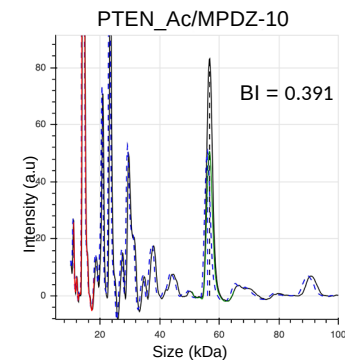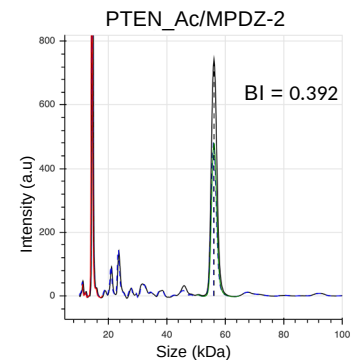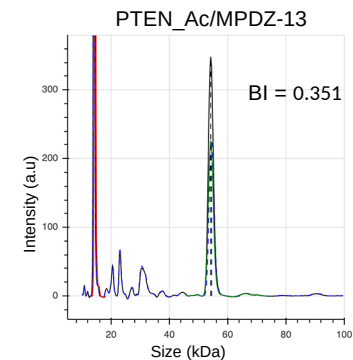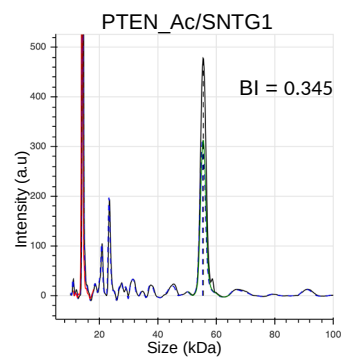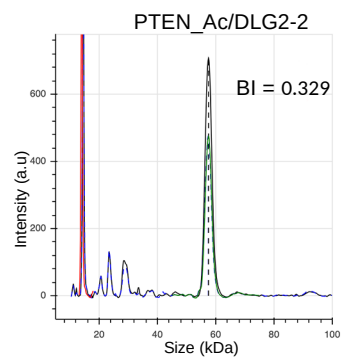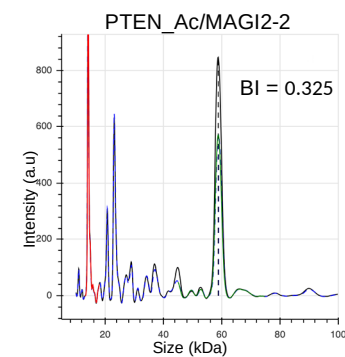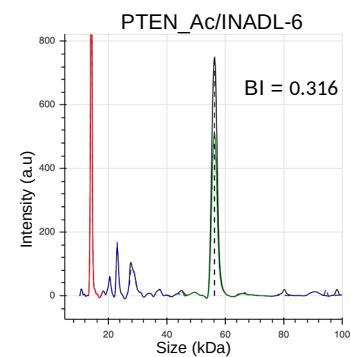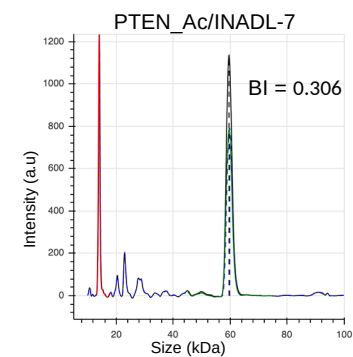

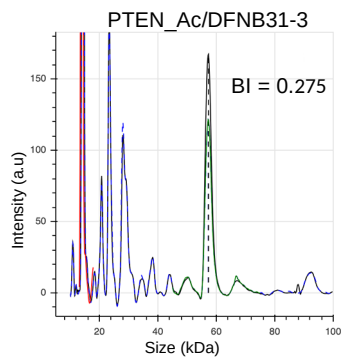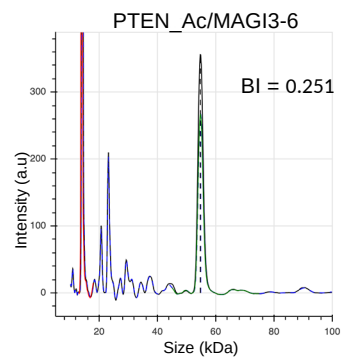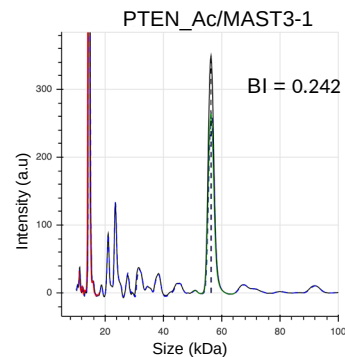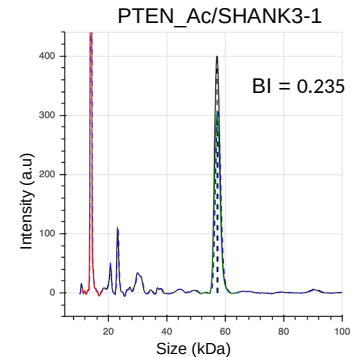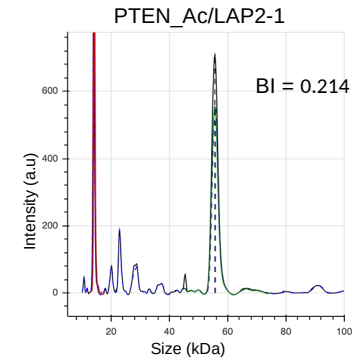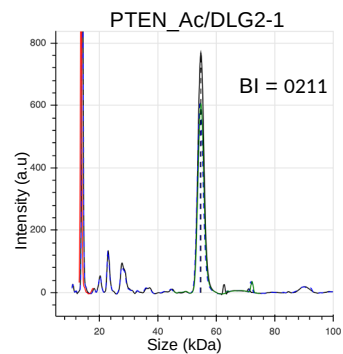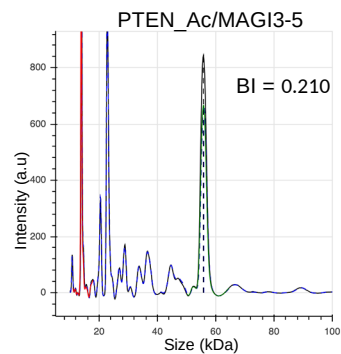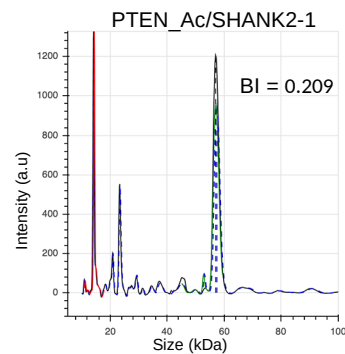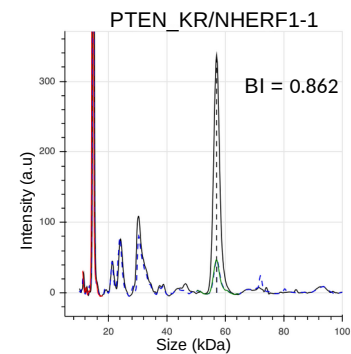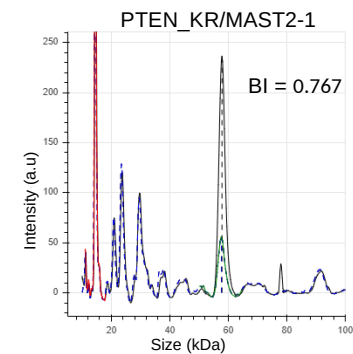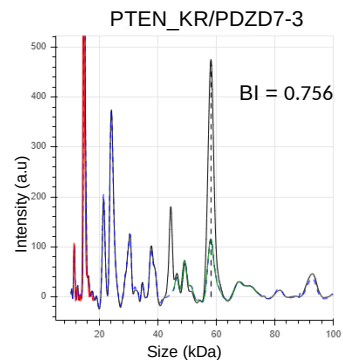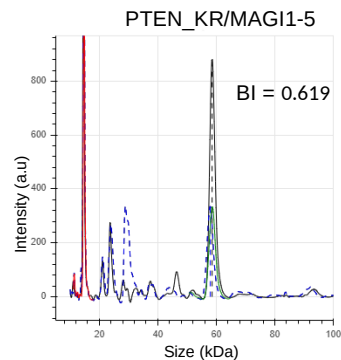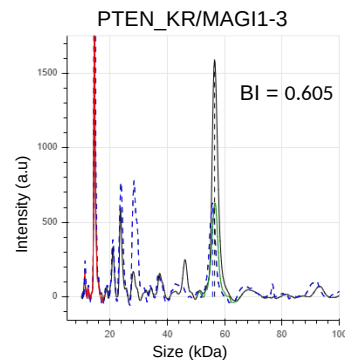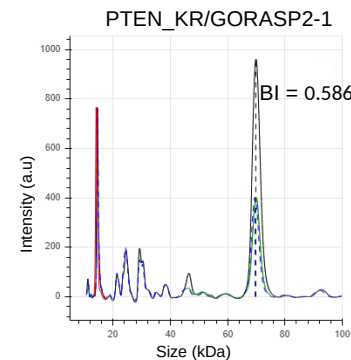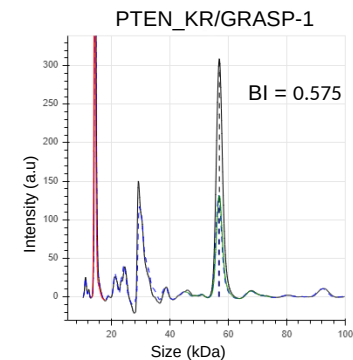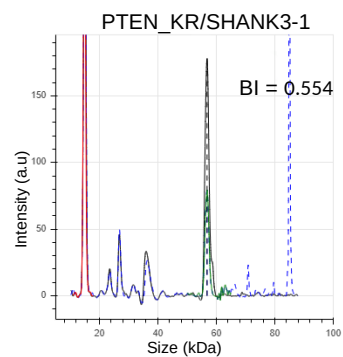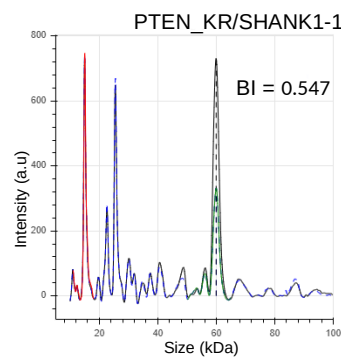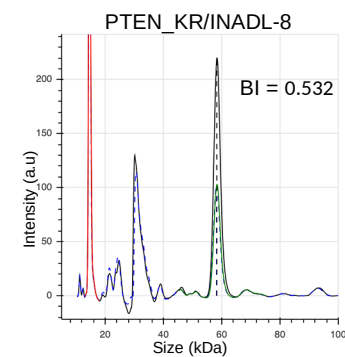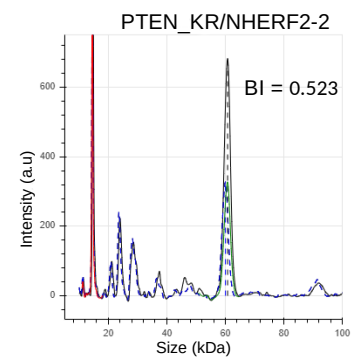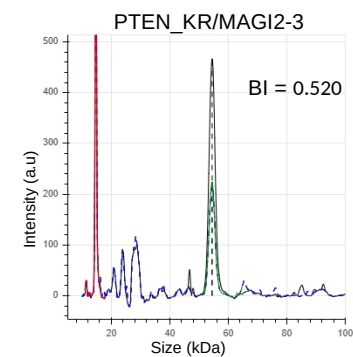

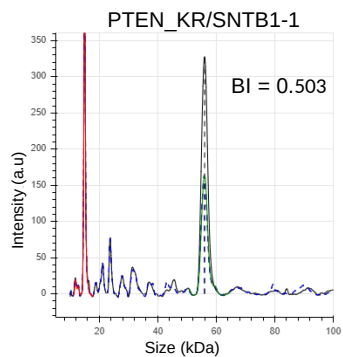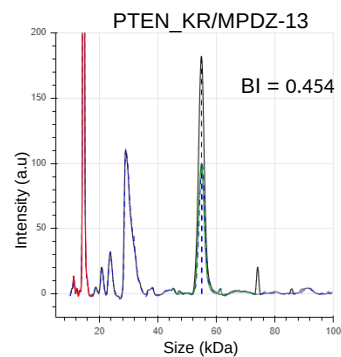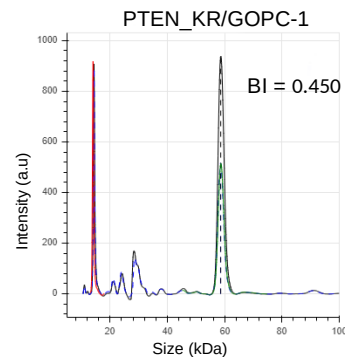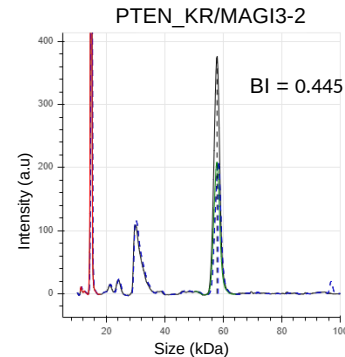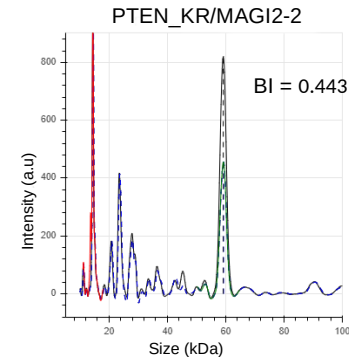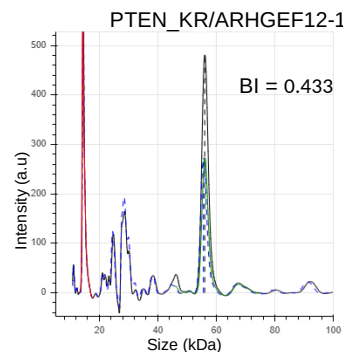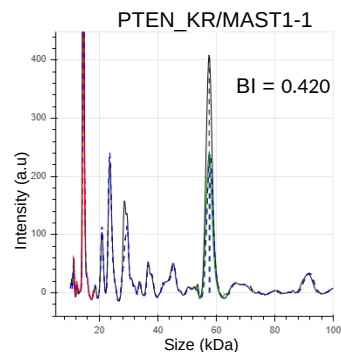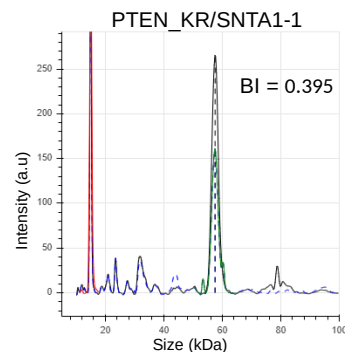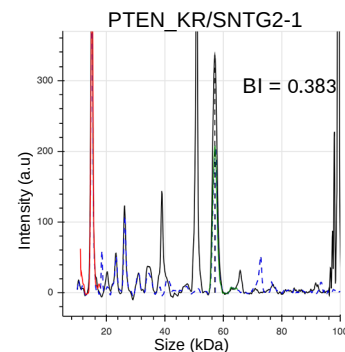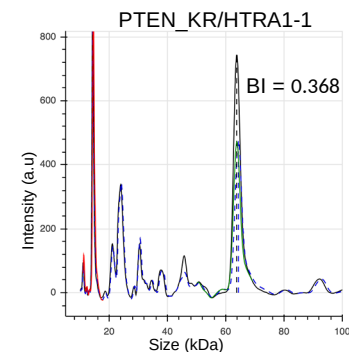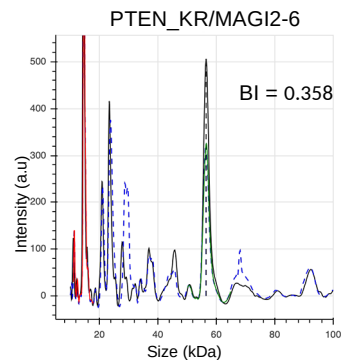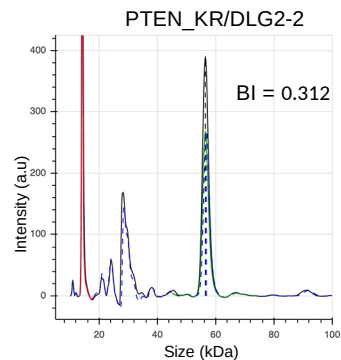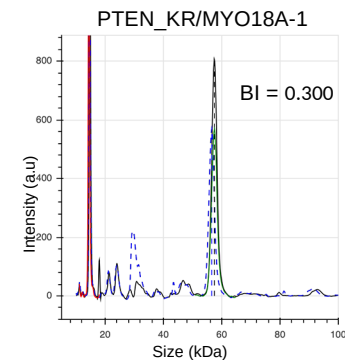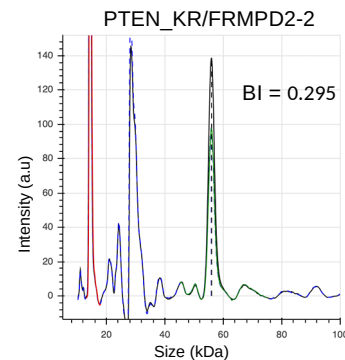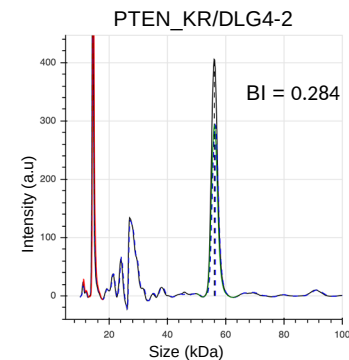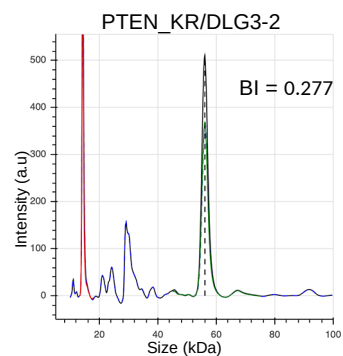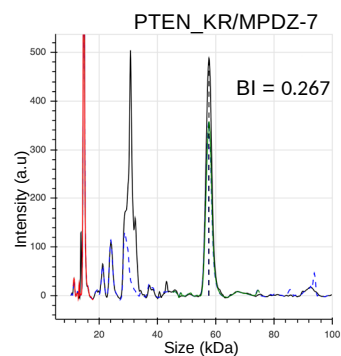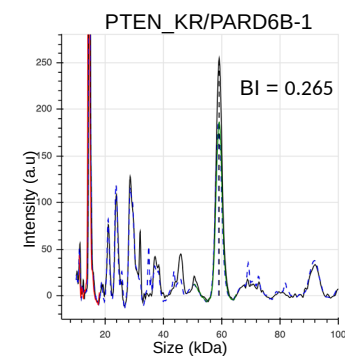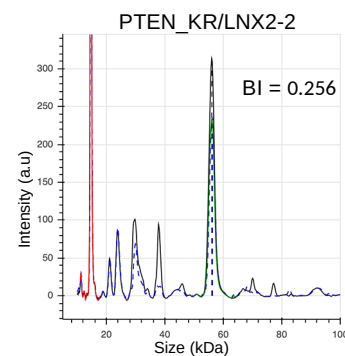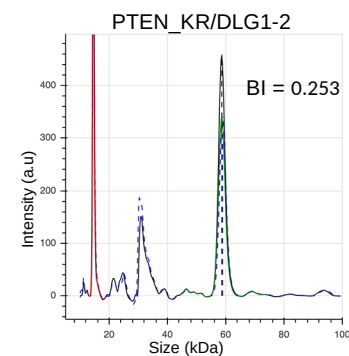

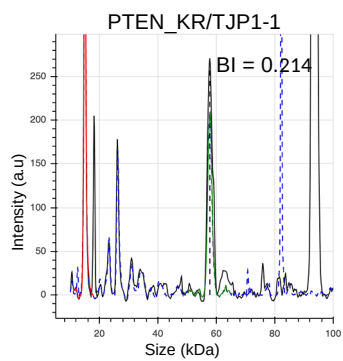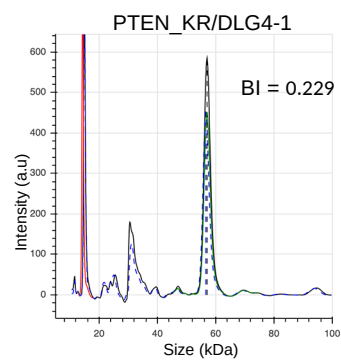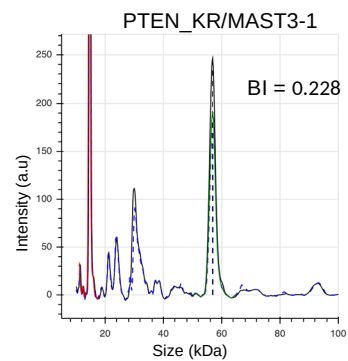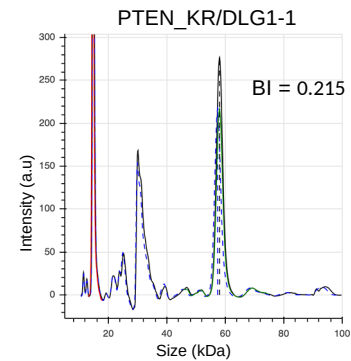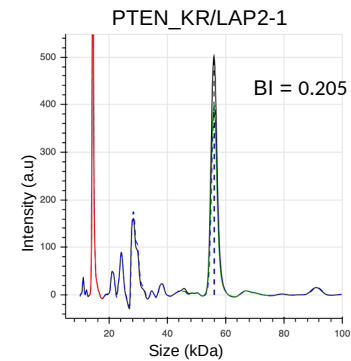

Supplement: S1 Fig — For each panel, after superimposition of the two electropherograms recorded for the PBM of interest (blue dotted line) and for the biotin reference (black solid line), the normalization of the PBM electropherogram compared to the reference one is done using the signal of the lysozyme added in every sample at a constant concentration (red peak). The region between 20 and 60 kDa which contains peaks of the crude extract supposedly to be constant, is used to verify the proper intensity normalization of the two electropherograms. The intensities of the peak of interest after proper alignment along the molecular weight scale (region covered by the green dotted line) are subsequently used to quantify the depletion of an individual PDZ domain and then the BI value. All those normalization and alignment steps are performed automatically. (PDF) [file pone.0244613.s001.pdf]

Supp. Fig. S2

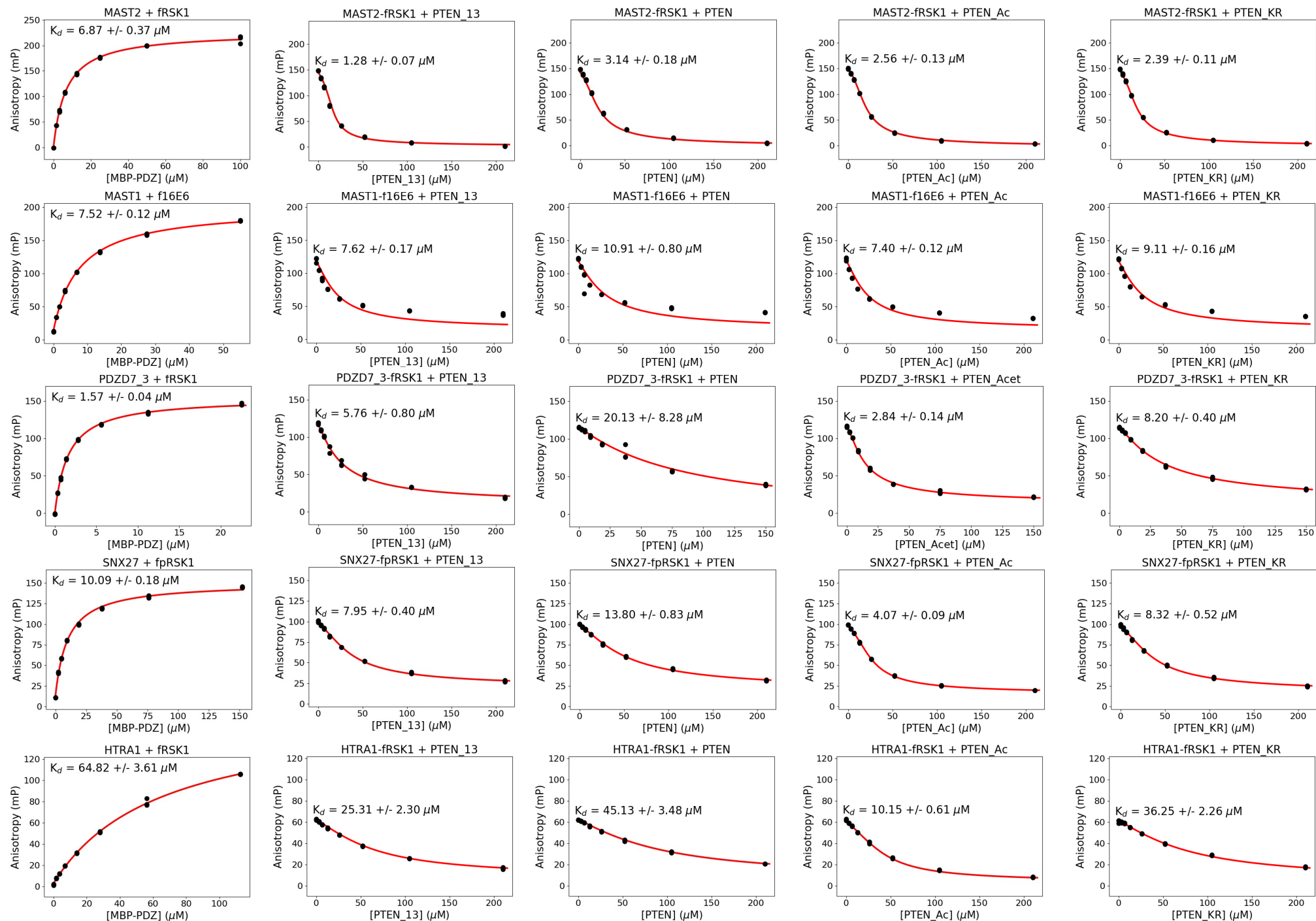

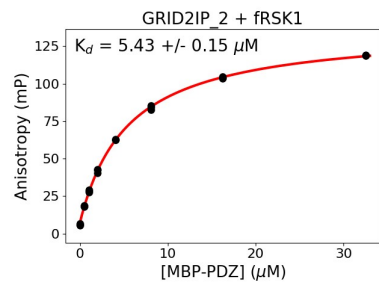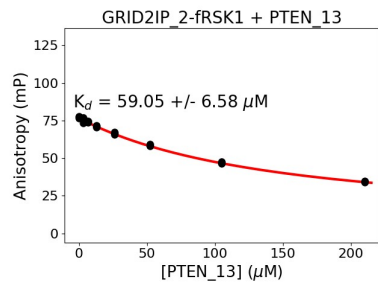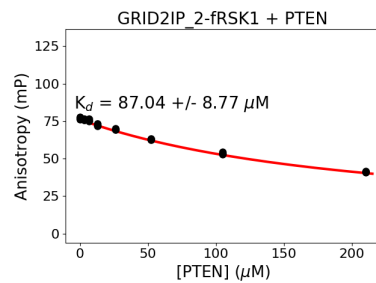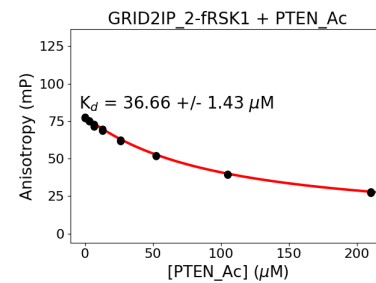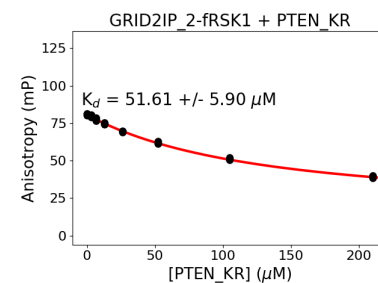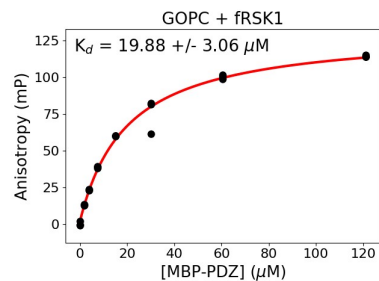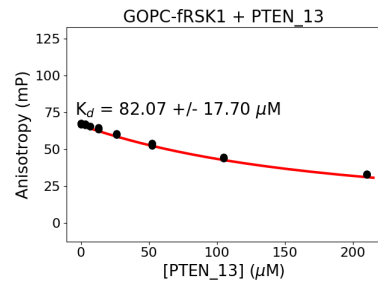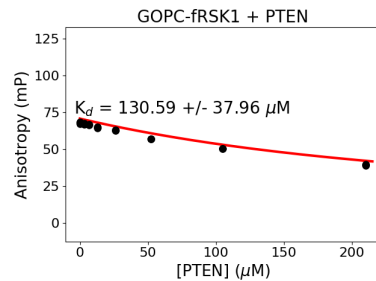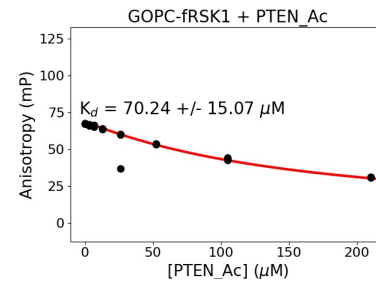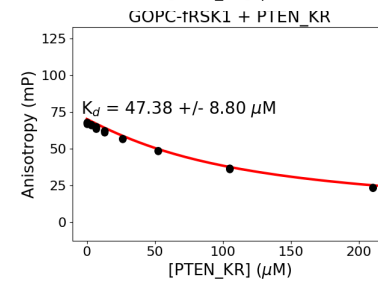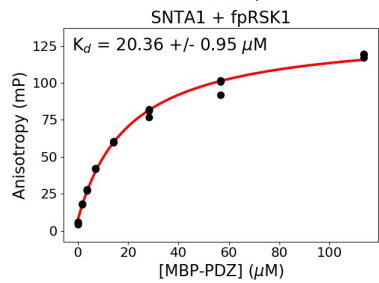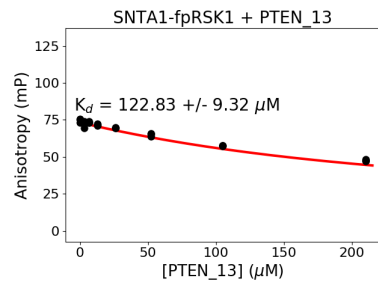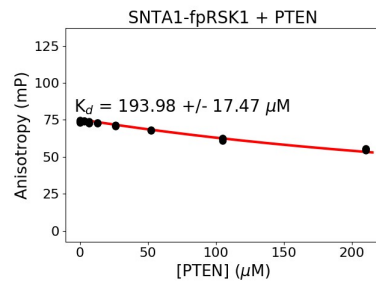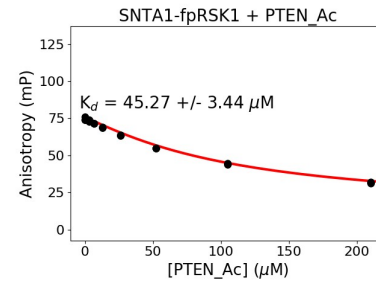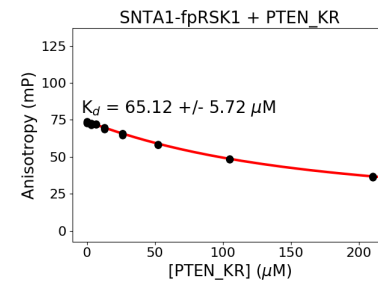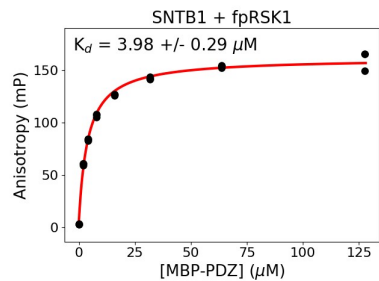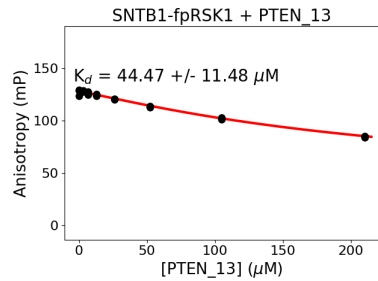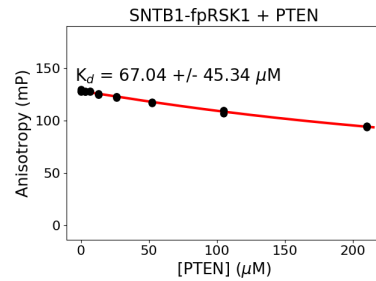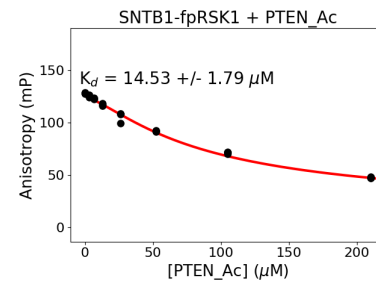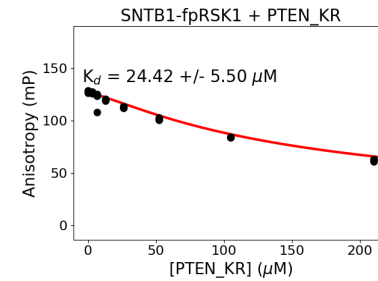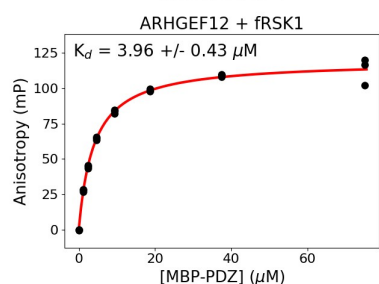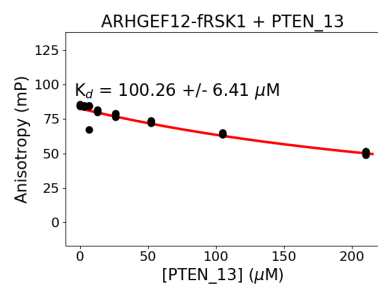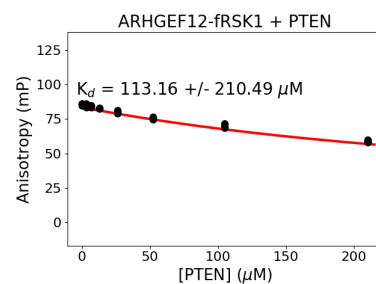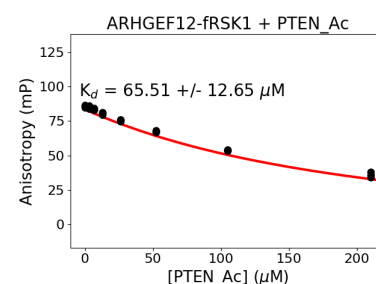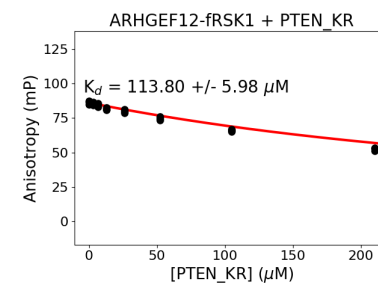

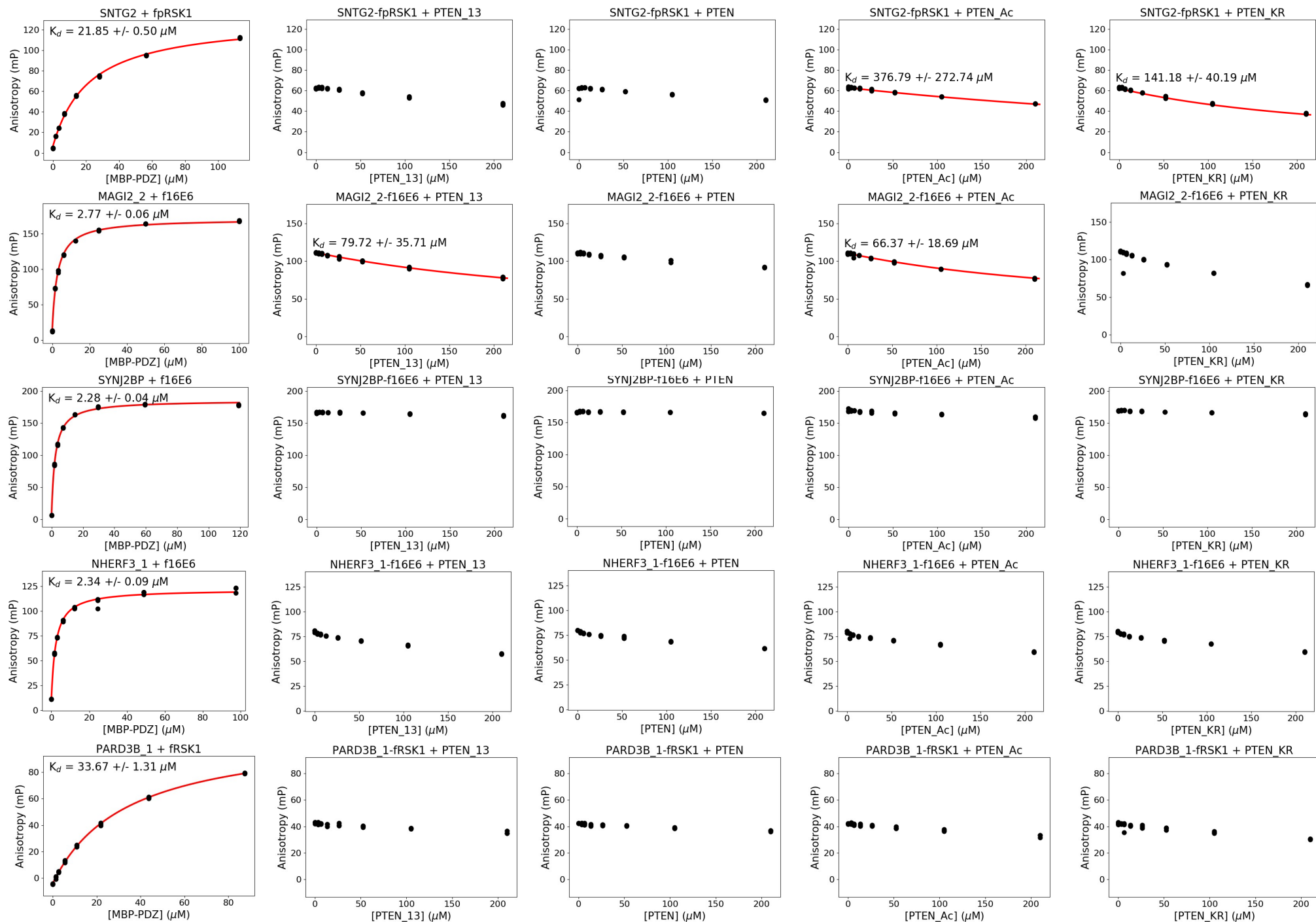

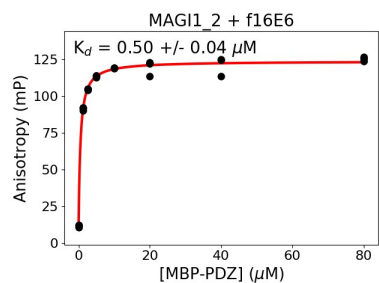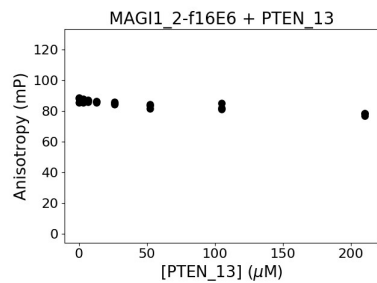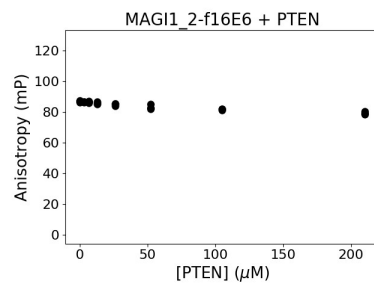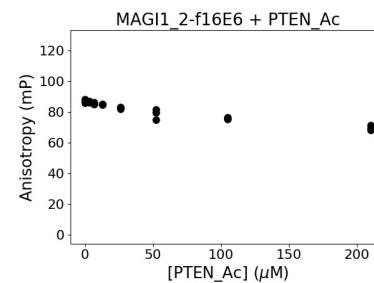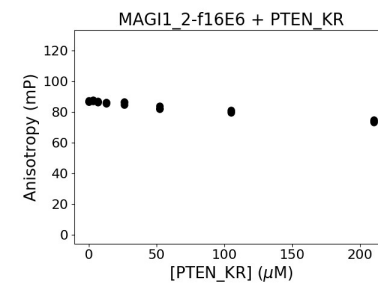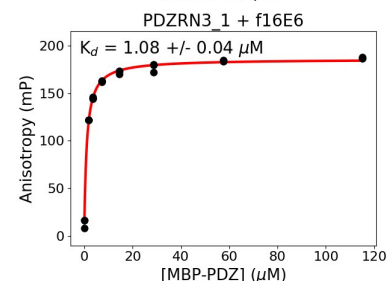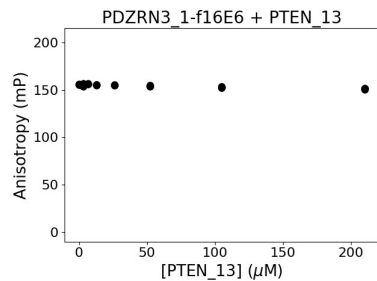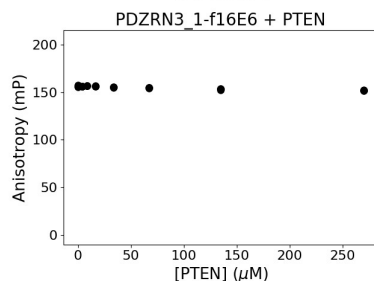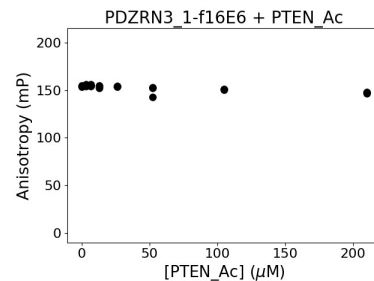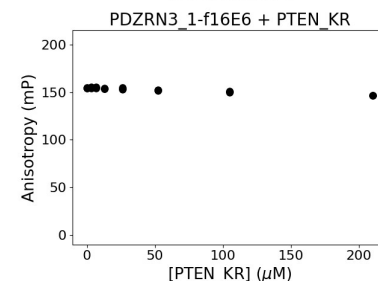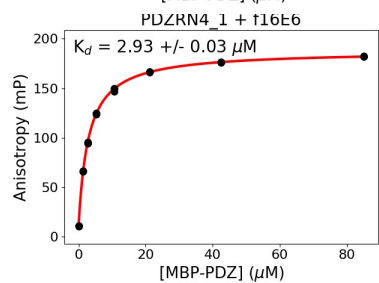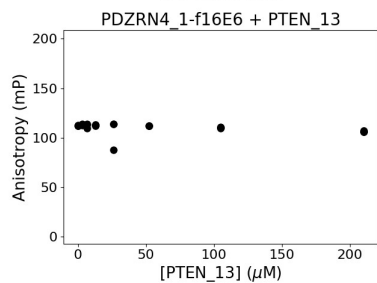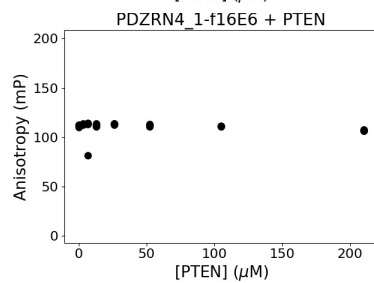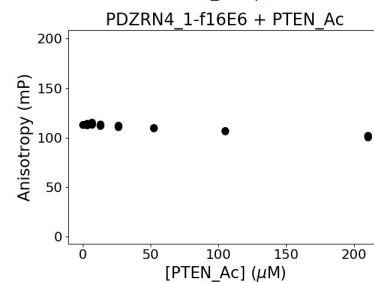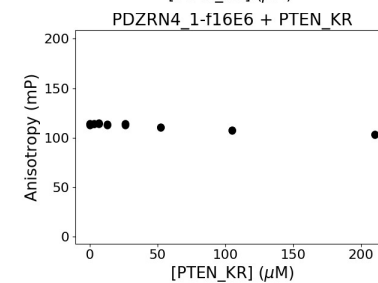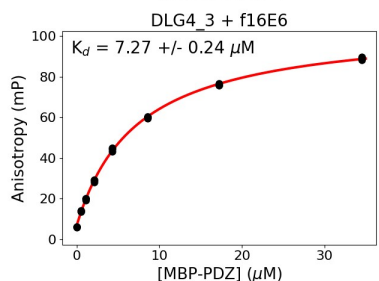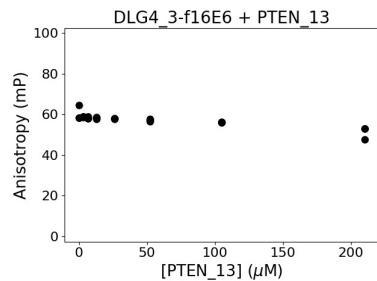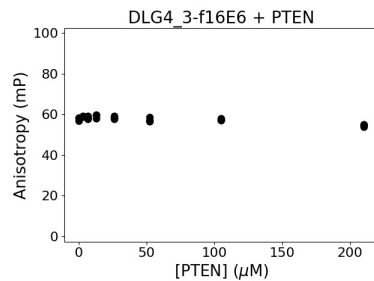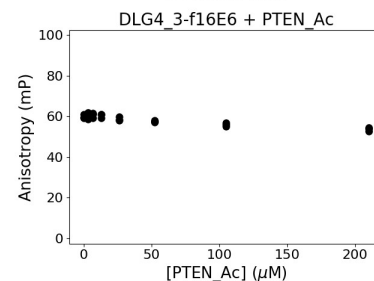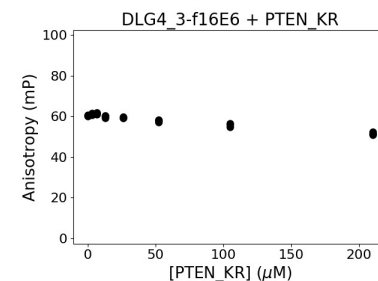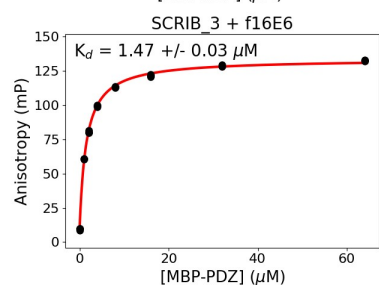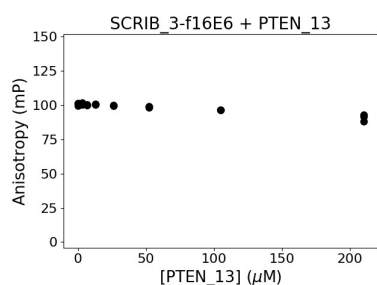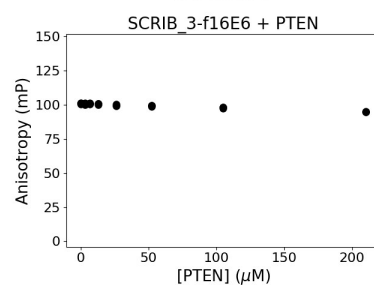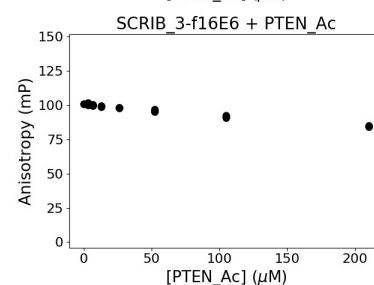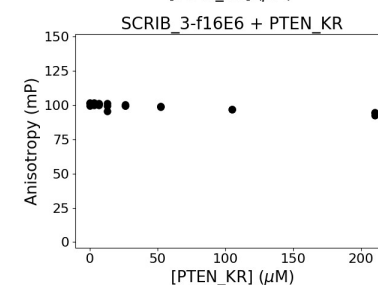

Supplement: S2 Fig — The first column contains direct FP data, while the others contain competitive FP data. FP data recorded in triplicate are represented by black dots. The reported dissociation constants and errors are the averages and the standard deviations of the fit (solid red curves) of 500 independent Monte-Carlo simulations, calculated using ProFit as described in Simon et al., 2020. (PDF) [file pone.0244613.s002.pdf]

Supp. Fig. S3

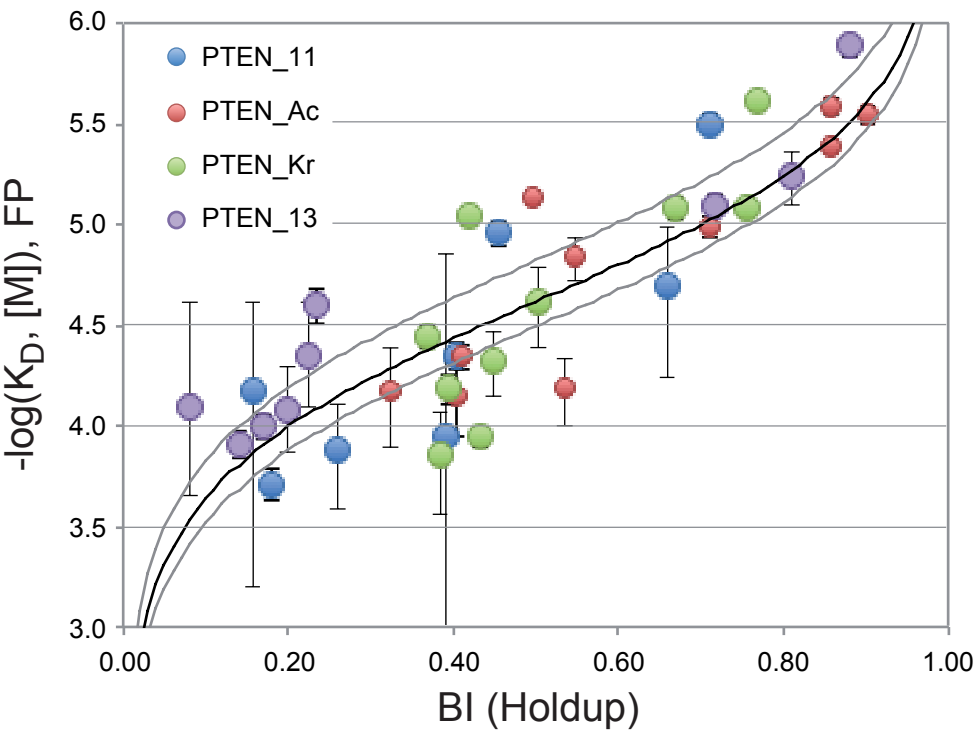

Supplement: S3 Fig — The scatter plot corresponds to experimental holdup BI KD_FP values colored according to the PBM peptides. Is superimposed the curves KD = f(BI) obtained from Eq 2 considering the global average peptide concentration (26 μM; black solid line) or the lower and upper peptide concentrations (17 and 34 μM, gray solid lines). Error bars are representative of peptide concentration uncertainties (See Fig 4) after their propagation into the–log(KD) values. (PDF) [file pone.0244613.s003.pdf]
