# Supplementary material for: Interactomic affinity profiling by holdup assay: Acetylation and distal residues impact the PDZome-binding specificity of PTEN phosphatase
Source: PLoS One. 2020 Dec 31;15(12):e0244613. doi: 10.1371/journal.pone.0244613 (PMC7774954; doi:10.1371/journal.pone.0244613)
Supplement: S2 File — (DOCX) [file pone.0244613.s005.docx]

**Supporting information**

**Interactomic affinity profiling by holdup assay: acetylation and distal residues impact the PDZome-binding specificity of PTEN phosphatase**

Pau Jané, Gergő Gógl, Camille Kostmann, Goran Bich, Virginie Girault, Célia Caillet-Saguy, Pascal Eberling, Renaud Vincentelli, Nicolas Wolff, Gilles Travé and Yves Nominé

**Supp. Fig. S1**: **the entire data set obtained by holdup for BI>0.20.** For each panel, after superimposition of the two electropherograms recorded for the PBM of interest (blue dotted line) and for the biotin reference (black solid line), the normalization of the PBM electropherogram compared to the reference one is done using the signal of the lysozyme added in every sample at a constant concentration (red peak). The region between 20 and 60 kDa which contains peaks of the crude extract supposedly to be constant, is used to verify the proper intensity normalization of the two electropherograms. The intensities of the peak of interest after proper alignment along the molecular weight scale (region covered by the green dotted line) are subsequently used to quantify the depletion of an individual PDZ domain and then the BI value. All those normalization and alignment steps are performed automatically.

**Supp. Fig. S2**: **the entire data set obtained by competitive FP.** The first column contains direct FP data, while the others contain competitive FP data. FP data recorded in triplicate are represented by black dots. The reported dissociation constants and errors are the averages and the standard deviations of the fit (solid red curves) of 500 independent Monte-Carlo simulations, calculated using ProFit as described in Simon et al., 2020.

**Supp. Fig. S3: comparaison of holdup BI and K_D_ values obtained by competitive FP**. The scatter plot corresponds to experimental holdup BI K_D__FP values colored according to the PBM peptides. Is superimposed the curves K_D_ = *f*(BI) obtained from **Eq. 2** considering the global average peptide concentration (26 µM; black solid line) or the lower and upper peptide concentrations (17 and 34 µM, gray solid lines). Error bars are representative of peptide concentration uncertainties (See Fig. 4) after their propagation into the –log(K_D_) values.

**Supp. Info S1** (Excel file): **all data set with all the BI values together with the transformed dissociation equilibrium constants for each PDZ-PBM interaction.** All the plots in the present study are obtained according to the data contained in this file.

**I_S_ uncertainty estimate**

Uncertainty on I_S_ was estimated according to several steps described below:

- Since it’s not possible strictly talking to propagate uncertainty for data recorded in singlicate, we considered that log(KD) uncertainty is contained in the correlation plot [log(KD,HU) vs. log(KD,FP)] when comparing the experimental data with the perfect theoretical correlation (the dotted diagonal line). Therefore we estimated the uncertainty on log(KD) scale by calculating the average of all the differences between the two log(KD) values for each PDZ and dividing it by a factor of 2.

- Originally, we estimated the 1/2 PDZ index by counting the number of PDZ with a log(KD) value above the threshold calculated with the formulae developed in the manuscript. Then we did the same type of counting for [threshold value + log(KD) uncertainty] and for [threshold value - log(KD) uncertainty].

- From the difference in the two counts is estimated the index uncertainty.

Due to the low number of PDZ occurrences, a Fisher's exact test was preferred to estimate the p-value. Calculations were performed online with <http://astatsa.com> using default parameters.

**References**

Simon M.A., Ecsédi P., Kovács G.M., Póti Á.L., Reményi A., Kardos J., Gógl G., & Nyitray L. (2019) High-throughput competitive fluorescence polarization assay reveals functional redundancy in the S100 protein family. *FEBS J.* 1–13.
